# Prognosis of colorectal cancer, prognostic index of immunogenic cell death associated genes in response to immunotherapy, and potential therapeutic effects of ferroptosis inducers

**DOI:** 10.3389/fimmu.2024.1458270

**Published:** 2024-09-20

**Authors:** Mengjie Lei, Meihua Xiao, Zhiqing Long, Taolin Lin, Ran Ding, Qi Quan

**Affiliations:** ^1^ State Key Laboratory of Oncology in South China, Collaborative Innovation Centre for Cancer Medicine, Sun Yat-sen University Cancer Center, Guangzhou, China; ^2^ Institute of Clinical Medicine, The First Affiliated Hospital of University of South China, Hengyang, Hunan, China; ^3^ Department of Radiology, State Key Laboratory of Oncology in South China, Guangdong Provincial Clinical Research Center for Cancer, Sun Yat-sen University Cancer Center, Guangzhou, China; ^4^ School of Biomedical Sciences and Engineering, South China University of Technology, Guangzhou, China; ^5^ Department of the VIP Region, Sun Yat-sen University Cancer Center, Guangzhou, China

**Keywords:** immunogenic cell death (ICD), colorectal cancer (CRC), ferroptosis inducers, combinatorial therapies, DAMPs, damage-associated molecular patterns

## Abstract

**Introduction:**

This study leverages bioinformatics and medical big data to integrate datasets from the Gene Expression Omnibus (GEO) and The Cancer Genome Atlas (TCGA), providing a comprehensive overview of immunogenic cell death (ICD)-related gene expression in colorectal cancer (CRC). The research aims to elucidate the molecular pathways and gene networks associated with ICD in CRC, with a focus on the therapeutic potential of cell death inducers, including ferroptosis agents, and their implications for precision medicine.

**Methods:**

We conducted differential expression analysis and utilized advanced bioinformatic techniques to analyze ICD-related gene expression in CRC tissues. Unsupervised consensus clustering was applied to categorize CRC patients into distinct ICD-associated subtypes, followed by an in-depth immune microenvironment analysis and single-cell RNA sequencing to investigate immune responses and cell infiltration patterns. Experimental validation was performed to assess the impact of cell death inducers on ICD gene expression and their interaction with ferroptosis inducers in combination with other clinical drugs.

**Results:**

Distinct ICD gene expression profiles were identified in CRC tissues, revealing molecular pathways and intricate gene networks. Unsupervised consensus clustering refined the CRC cohort into unique ICD-associated subtypes, each characterized by distinct clinical and immunological features. Immune microenvironment analysis and single-cell RNA sequencing revealed significant variations in immune responses and cell infiltration patterns across these subtypes. Experimental validation confirmed that cell death inducers directly affect ICD gene expression, highlighting their therapeutic potential. Additionally, combinatorial therapies with ferroptosis inducers and clinical drugs were shown to influence drug sensitivity and resistance in CRC.

**Discussion:**

Our findings underscore the importance of ICD-related genes in CRC prognosis and therapeutic targeting. The study provides actionable insights into the efficacy of cell death-inducing therapies, particularly ferroptosis inducers, and their regulatory mechanisms in CRC. These discoveries support the development of precision medicine strategies targeting ICD genes and offer valuable guidance for translating these therapies into clinical practice, with the potential to enhance CRC treatment outcomes and patient survival.

## Graphical Abstract

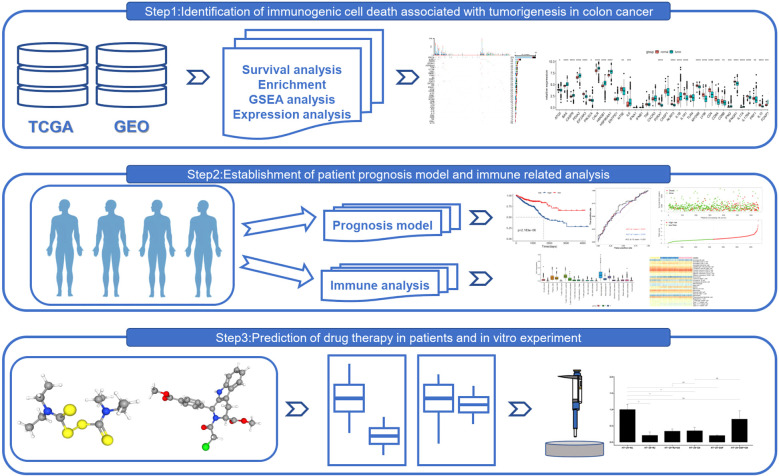

## Introduction

Unlike proinflammatory necrosis, apoptosis has traditionally been regarded as a form of acceptable cell death. It is currently established that damage-associated molecular patterns (DAMPs), which are released after the apoptosis of cancerous or diseased cells, can trigger both innate and adaptive immune responses. Immunogenic cell death (ICD), which is triggered by the release of DAMPs, can result in the generation of tumor-specific CD8+ T cells and immunological memory (1).

ICD seems like a potential way to treat cancer, but as of right now, only a small number of tumor therapeutics—including radiation, oncolytic virotherapy, oxaliplatin, cetuximab, bortezomib, photodynamic therapy, and extracorporeal photochemotherapy—are known to cause ICD. These medications encourage alterations in the extracellular milieu or cell surface that are consistent with the induction of ICD (2, 3). The avoidance of immunological destruction, which can be accomplished by secreting immunosuppressive substances, enlisting immunosuppressive cells as T regulatory cells, and downregulating the activation of cytotoxic lymphocytes, is one of the primary characteristics of cancer as outlined by Hanahan and Weinberg in 2011 (4). In this context, the natural history of a number of solid malignancies, such as melanoma, lung cancer, and urological tumors, has changed due to the use of immune checkpoint inhibitors (ICIs) (5, 6). Antigen-presenting cells (APCs) and cancer cells, in fact, express ligands on their plasma membranes that bind to immunological checkpoints on lymphocytes and prevent their activation; ICIs disrupt this process and allow T-cell function to be restored (6). The relationship between ICIs and ICD inducers and their possible function in inducing long-lasting immune responses is poorly understood. This could ultimately lead to cancer vaccination, particularly in diseases like gastrointestinal (GI) cancer where the benefits of immunotherapy alone are not very great (7). With 3.4 million cancer deaths from the disease in 2018 and nearly 26% of all cancer cases worldwide, gastrointestinal cancer is a serious problem (8).

Zheng’s study highlights the ways in which ferroptotic colon cancer cells and antitumor immunity work in concert by boosting immunogenicity, specifically the production of HMGB1 and the exposure of CRT (9, 10). In addition to accelerating DC maturation, these cells also enhance the immunological milieu by raising the frequency of antitumor immune cells and T cell release of IFN-γ (11). Crucially, immune checkpoint inhibitors (ICIs) become much more effective when ferroptosis is induced, and this improvement is reliant on antitumor immunity being activated (12). The study also discovered that while certain cells, including MDSCs, are resistant to ferroptosis, neutral ceramidase N-acylsphingosine amidohydrolase can be inhibited to overcome this resistance (13). Immune cells, such as CD8+ T cells, collaborate with arachidonic acid to induce iron poisoning in cancer cells (14).

These results demonstrate the potential of ferroptosis in colon cancer immunotherapy and offer novel therapeutic approaches for future ferroptosis-targeted immunotherapy combinations (14). The identification of treatment-related targets and the investigation of the activating effects of ferroptosis inducers on immunogenic cell death and antitumor immunity are necessary because the therapeutic mechanism of targeting immunogenic cell death-related genes in tumors through the ferroptosis mechanism of tumor cells remains unknown (11).

## Materials and methods

### Data download and processing

RNA-seq data, related clinical data, and survival data for colorectal cancer were downloaded from the UCSC-Xena platform. Included in this were count numbers from the TCGA-COAD and TCGA-READ data sets, which the TCGAbiolinks package was used to standardize and process further (15). To be more precise, the sample bearing the ID “-11A” was classified as neighboring normal tissue, whilst the remaining samples were identified as malignant tissue. Of them, 689 samples containing prognostic data—51 normal tissue samples and 638 malignant tissue samples—were used in the creation and examination of the models that followed.

### Gene-expression data processing

Initially, we concentrated on creating a list of genes that were closely linked to immunogenic cell death (ICD) in order to better understand the molecular mechanisms underlying this phenomenon. These genes, which have been chosen from previous research, serve as the cornerstone of our ICD investigation. We then successfully created a gene expression matrix specifically for ICD by using differential analysis techniques to match these genes with expression profiling data from tumor and normal tissues. The primary dataset for our ensuing analyses is this matrix. The exact roles of ICD-related genes in tumor development were determined by using the Wilcoxon rank-sum test to the ICD gene expression data from tumor and normal tissue samples in the TCGA database. The study’s findings demonstrated that there were differences in the patterns of these genes’ expression between the two sets of samples. Furthermore, Pearson correlation analysis was used to explore potential interactions between these genes, revealing expression connections that improved our understanding of the ICD gene network. We used the STRINGdb program to build a protein-protein interaction (PPI) network based on known protein interactions for ICD-related genes in order to reveal the molecular processes of ICD at the protein level (16). This network offers insights into the intricate processes involved in ICD by visualizing important proteins and their connections. Lastly, we used the clusterProfiler tool to conduct pathway enrichment analysis in order to obtain a better understanding of the possible roles that these genes may play in biological processes. Significant enrichments of ICD-related genes were found in particular biological pathways by this study, suggesting that these genes may play roles in immune response, tumor growth, and other biological processes. These thorough findings set a strong basis for further study in this area and offer vital insights on the function of ICD in tumor development.

### Unsupervised clustering analysis identified ICDRGs expression patterns

To gain a deeper understanding of the molecular characteristics and subtype prediction of CRC, we employed an approach focused on the differential expression of ICD-related genes. Utilizing the “ConsensusClusterPlus” package (version 1.62.0), we performed unsupervised clustering analysis on CRC patients, setting the parameters maxK to “6”, clusterAlg to “hc” (hierarchical clustering), and distance to “Pearson”. During this process (17), we identified the optimal number of immune subtypes (K) by tracking cells, analyzing cumulative distribution functions, and their relative changes. Subsequently, we collected and analyzed sample information from different clinical subgroups (such as age, stage, TNM classification, and survival status) within each subtype. To visually present this information, we leveraged the tidyverse (version 1.3.2) and ggplot2 (version 3.3.6) packages to visualize the classification information of different subtypes across various subgroups (18, 19). In the subtype analysis phase, we utilized the TCGA dataset and employed the Kaplan–Meier curve method to assess the correlation between ICD-related gene consensus clustering subtypes and survival outcomes, based on subtype classification, patient survival time, and status. Additionally, we generated a heatmap of gene expression, revealing the relationship between gene expression, subtype classification, and clinical characteristics by observing the expression values of genes in TCGA samples and the clinical features of each sample. This integrated analysis not only provided us with a profound understanding of CRC molecular subtypes but also highlighted the potential value of ICD-related genes in predicting patient prognosis and guiding treatment. These findings offer new strategies and insights for precision medicine in CRC, paving the way for further research and clinical applications.

### Analysis of immune microenvironment differences between subtypes

In this study, we employed two advanced bioinformatics algorithms to extensively profile the immune microenvironment of colorectal cancer samples from The Cancer Genome Atlas (TCGA). The application of these methods not only sheds light on the intricate mechanisms of tumor immune response but also provides crucial scientific evidence for future clinical diagnosis and therapeutic strategies.

Firstly, we leveraged the CIBERSORT algorithm (http://cibersort.stanford.edu/index.php), a robust tool based on linear support vector machine (SVM) principles, to accurately deconvolve the expression matrix of immune cell subtypes in tumor samples (20). Through both relative and absolute modes of computation, we determined the composition of 22 immune cell types within the samples. The relative mode ensures that the proportions of different immune cell subtypes sum to one, reflecting their relative contributions to the overall immune response. Meanwhile, the absolute mode directly provides the absolute counts of each immune cell type, offering a more intuitive understanding of their actual roles in immune responses. Furthermore, we utilized the Wilcoxon rank-sum test to analyze the differences in the proportions of various immune cell subtypes and calculated the corresponding statistical significance (p-values). Additionally, we plotted boxplots based on the subgrouping of immune cell types to visually demonstrate these differences.

Secondly, to assess the enrichment of different immune gene sets in tumor samples, we employed the ssGSEA (2.0) algorithm to evaluate 17 immune gene sets from the import database (21). This approach comprehensively reflects the overall expression levels of gene sets in samples, thus uncovering their potential roles in immune responses. Specifically, we focused on the expression differences of HLA family genes among different immune cell subtypes, as these genes play a pivotal role in immune responses. By extracting the expression data of HLA family genes and combining it with the Wilcoxon rank-sum test, we compared the expression differences of immune checkpoint genes and HLA family genes between different immune cell subtypes and calculated the statistical significance (p-values) between high-risk and low-risk groups. Finally, we utilized the ggpubr package in R to plot boxplots, graphically representing these differences (22).

In summary, by integrating the CIBERSORT algorithm and the ssGSEA algorithm, we comprehensively and in-depth profiled the immune microenvironment of TCGA colorectal cancer samples. These findings not only enhance our understanding of the complex mechanisms of tumor immune response but also provide robust data support and scientific evidence for future clinical diagnosis and therapeutic strategies.

### Functional enrichment analysis of genes

Gene Ontology (GO) and Kyoto Encyclopedia of Genes and Genomes (KEGG) enrichment studies were conducted using several R packages, such as “org.Hs.Eg.Db,” “ggplot2,” and “clusterProfiler,” to investigate the biological processes enriched by the genes in key modules (23). Any GO or KEGG terms that showed significant enrichment were examined further with a p-value threshold of 0.05.

### Establishing a prognosis signature related to ICDGs

Further, utilizing the limma package in R language, which employs linear regression and empirical Bayes methods, we conducted differential expression analysis comparing tumor samples with normal samples across all TCGA colorectal cancer datasets. This analysis yielded gene-specific information such as P-values and logFC. Additionally, we applied the Benjamini & Hochberg method for multiple testing correction, resulting in adjusted P-values (adj.P.Value) (24). We evaluated the genes based on both fold-change and significance, setting thresholds as adj.P.Value < 0.05 and |logFC| > 1.

Incorporating the clinical survival and prognostic data from the TCGA colorectal cancer samples, we screened for genes significantly associated with overall survival prognosis using univariate Cox regression analysis from the survival package in R. Genes with a P-value less than 0.05 were considered significantly correlated.

LASSO Cox analysis identified the genes most relevant to overall survival. To prevent overfitting, we performed 10-fold cross-validation using the glmnet package in R. Based on RNA expression levels, we then calculated a risk score for each patient using the formula:


(1) $$Risk\;score=\;\sum_{i=1}^{n}coefi\;X\;id$$

where coefi represents the coefficient and Xi is the normalized count for each gene.

Using clinical follow-up data from the GSE17536 and GSE39582 colorectal cancer datasets, we computed the Risk score for each sample in both the TCGA training set and the GEO validation set. Samples were then divided into High (Risk score above the median) and Low (Risk score below or equal to the median) groups. The Kaplan-Meier curve method from the survival package was employed to assess the correlation between these groups and actual survival outcomes. This risk model was further validated for robustness by evaluating risk scores on an external independent dataset.

### Quality control and cell-type identification

For quality control, Seurat (version 4.3.0) was used to count unique molecular identifiers (UMIs) and mitochondrial genes. Cells with more than 100 UMIs and less than 15% mitochondrion-derived UMI counts were selected (25). This study selected the top 20 components and first 2000 variable genes. The “ScaleData” function was used to regress the inflow of UMIs and the percentage of mitochondrion-derived UMI counts. Subsequently, the main cell clusters were identified by Seurat’s “FindClusters” function. Unbiased cell type recognition was visualized by umap.

### Cell clusters annotation

To delineate the specific marker genes for individual cell clusters, we employed the ‘FindMarkers’ function in Seurat to contrast the gene expression profiles of cells within a given cluster against all other cells in the dataset. This function utilizes a Wilcoxon rank-sum test to identify differentially expressed genes (DEGs) between the two groups. Subsequently, the obtained P values were adjusted for multiple testing using Bonferroni correction, considering the total number of genes analyzed. Marker genes were defined as those with an adjusted P value below 0.05 and exhibiting at least a twofold higher average expression level in the cluster of interest compared to all other clusters. To validate the annotation of each cell cluster, we relied on the expression of canonical marker genes. Specifically, T/NK cells were identified by the expression of CD3D, CD3E, and NKG7; memory B cells were characterized by CD79A and MS4A1; plasma cells were defined by the presence of IGHG1, JCHAIN, and MZB1. Monocytes and macrophages were distinguished by CD68, CD163, CD14, and LYZ, while dendritic cells were identified through CD74, CLEC9A, and CD1C. Fibroblasts exhibited high expression of COL1A1, ACTA2, and TAGLN, while endothelial cells were marked by VWF, PLVAP, and CLDN5. Epithelial cells, on the other hand, were distinguished by EPCAM, KRT8, and KRT18. This comprehensive analysis, leveraging both statistical testing and the expression of established marker genes, allowed us to accurately annotate and characterize each cell cluster.

### Chemotherapy response analysis

Information on drug responsiveness and drug targeting pathways was gathered using the Cancer Drug Sensitivity Genomics (GDSC) platform. Subsequently, the pRRophetic package in R was employed to predict the drug sensitivity of various phenotypes from gene expression data, and to obtain the pharmaceuticals associated with different classes after tabulating the sensitivity data of the two GBM classes to various drugs, thereby setting the stage for the selection of clinical drugs (26).

### Quantitative reverse transcriptase PCR

The HCT-116(human colon cancer cell) lines were procured from ATCC. Following incubation for a duration of 12 to 24 hours, these cells were subjected to treatment with various compounds, including 2uM RSL3 (Selleck, catalog number #S8155), 0.25 μM disulfiram (Selleck, catalog number #S1680), and 20uM oxaliplatin (Selleck, catalog number S1224), for specified durations. Additionally, control groups were maintained without any drug exposure to serve as references. This experimental setup aimed to investigate the genome responses to these pharmacological agents.

Control groups were created by combining HCT-116 cells to compare the expression of MMP1, MMP3, MMP10, MMP12, CHST13, CXCL1, SERPINA1,SPINK4 WNT5A, between medication group and human colon cancer cell lines. The sequence of gene primers is shown in **Supplementary Table S1**. Total RNA was extracted using the Redzol kit (Beijing SBS Gene Technology Co., Ltd), and qRT-PCR was performed using the SYBR^®^ Premix Ex Taq™ II Kit. The relative mRNA expression levels were calculated using the 2−ΔΔCt method, with GAPDH as the internal reference gene.

### Distribution of cell subsets of treatment-related ICDRGs

Next, based on the experimental results, we collected three CRC single-cell profiles from the geographical dataset to further investigate the subgroup distribution of treatment-related ICDRGs in CRC. The GSE144735 dataset was derived from single-cell 3’ mRNA sequencing data of the tumor core, marginal zone, and normal mucosa from six Belgian patients. Low quality cells were screened by nUMI, nGene and mitochondrial gene ratio, and 27 414 high quality cells were finally selected. The GSE146771 dataset was subjected to single-cell RNA sequencing of multiple colorectal cancer patient samples using SMART-seq2 and 10x Genomics platforms. Different types of immune cells including myeloid cells, B cells, NK cells, T cells and non-immune cells were enriched by flow cytometry for CD45 staining and sorting. Smart-seq2 platform was used to sequence T cells from colorectal cancer patients in GSE108989 dataset. The classification includes CD8+ T cells, helper T cells, regulatory T cells, etc. Peripheral blood, normal intestinal mucosa, and tumor samples were subjected to cell sorting and sequencing. These datasets provide transcriptome data of multiple cell types in tumors and their microenvironment, which can help to investigate the diversity and specificity of tumor immune microenvironment.

### Statistical analysis

Survival analysis was conducted using the R survival package, and the Log-Rank test was utilized to assess the survival rates of each group. The Kruskal-Wallis test was employed to compare the differences between two or more sets of data, while the Wilcoxon test was used to compare the differences between the two groups. Kaplan-Meier technique was applied to generate survival curves for each subgroup within the dataset. The Spearman correlation analysis was used to determine the correlation coefficient. Furthermore, protein-protein interactions were analyzed using the string database to gain insights into how protein-coding genes interact with one another. Statistical significance was determined at P<0.05 for all calculations, which were performed using R versions 4.1.0 and 4.0.0.

## Results

### Expression levels of ICDRGs in CRC

**Figure 1:**
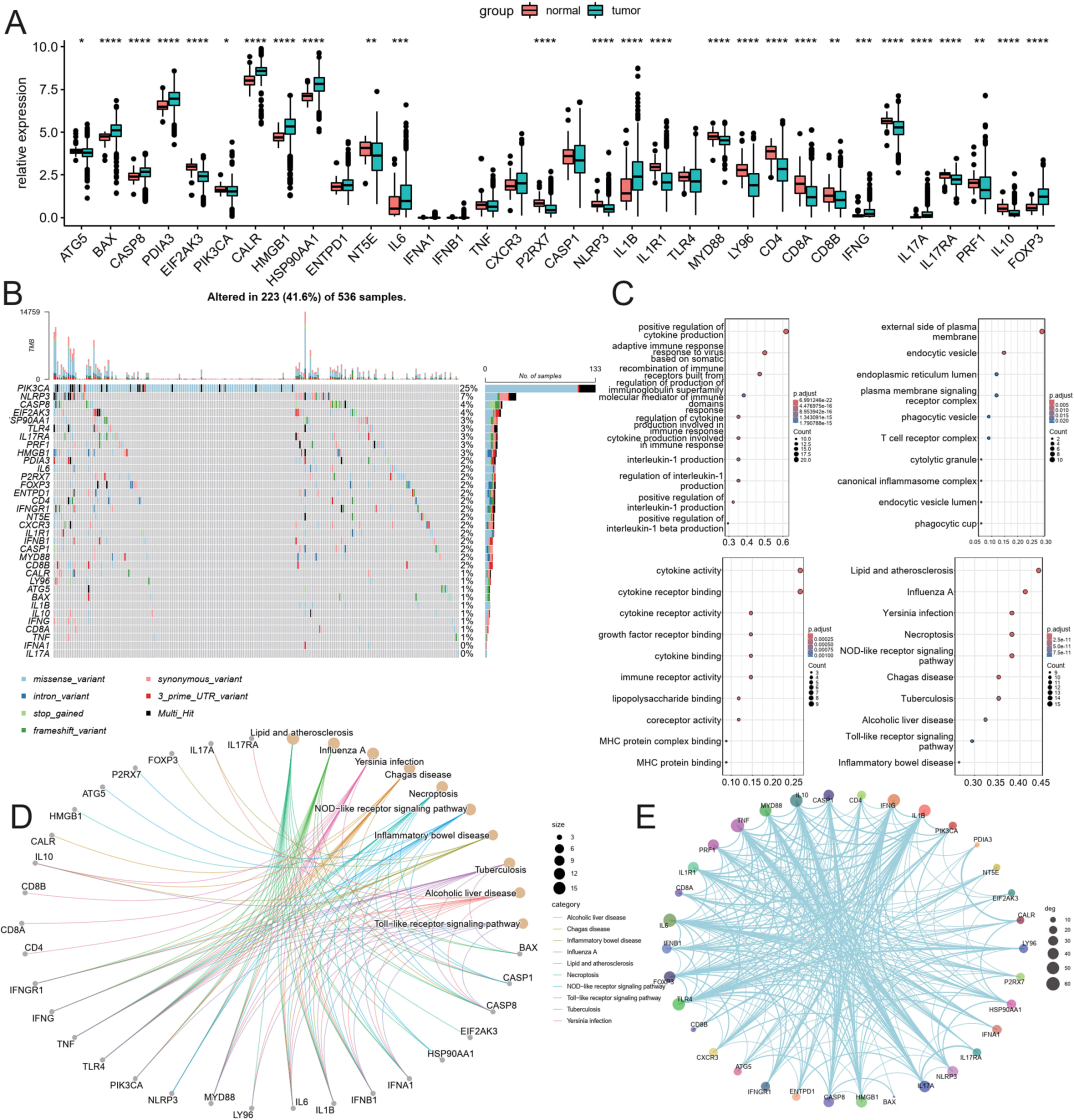
Analysis of ICDRGs expression. **(A)** Box plot of ICDRGS expression in tumor and normal CRC samples. **(B)** Mutations landscape of ICDRGS. **(C)** GO enrichment and pathway analysis of ICDRGs: Biological processes, cellular components, molecular functions. **(D)** KEGG pathway analysis. **(E)** Protein and protein interaction analysis.* <0.05, ** <0.01, *** <0.001 and **** <0.0001.

Transcriptomes and clinical data for 504 normal and colorectal cancer tissue samples were obtained from the TCGA database. This study encompasses 452 colorectal cancer patients with both clinical information and gene expression profiles. The expression of 34 ICDRGs was assessed using the Wilcoxon test, resulting in the identification of 26 ICDRGs with high tumor expression (|log2(FC)|, Pvalue < 0.05). The expression correlation of these ICDRGs was examined based on their tumor expression levels (**Figure 1A**). In the TCGA immunogenicity cell death-related gene mutation analysis, PI3KA, NLRP3, CASP8, EIF2AK3, HSP90AA1K, TLR4, IL17RA, PRF1, HMGB1 mutation frequency is higher (**Figure 1B**). Further, we analyzed the role of these ICDRGs in biological pathways, biological processes, cellular components, and molecular functions (**Figures 1C–E**). Biological pathways are more enriched in diseases prone to inflammatory response, as well as NOD−like receptor signaling pathway, and Toll−like receptor signaling pathway. Biological processes mainly focus on positive regulation of cytokine production and adaptive immune response based on somatic cells, etc. The cell components mainly concentrated on the external side of the plasma membrane, endocytic vesicle, and so on. The molecular functions mainly focus on cytokine activity, cytokine receptor binding, and so on.

### Development of the new CRC subtyping method

**Figure 2:**
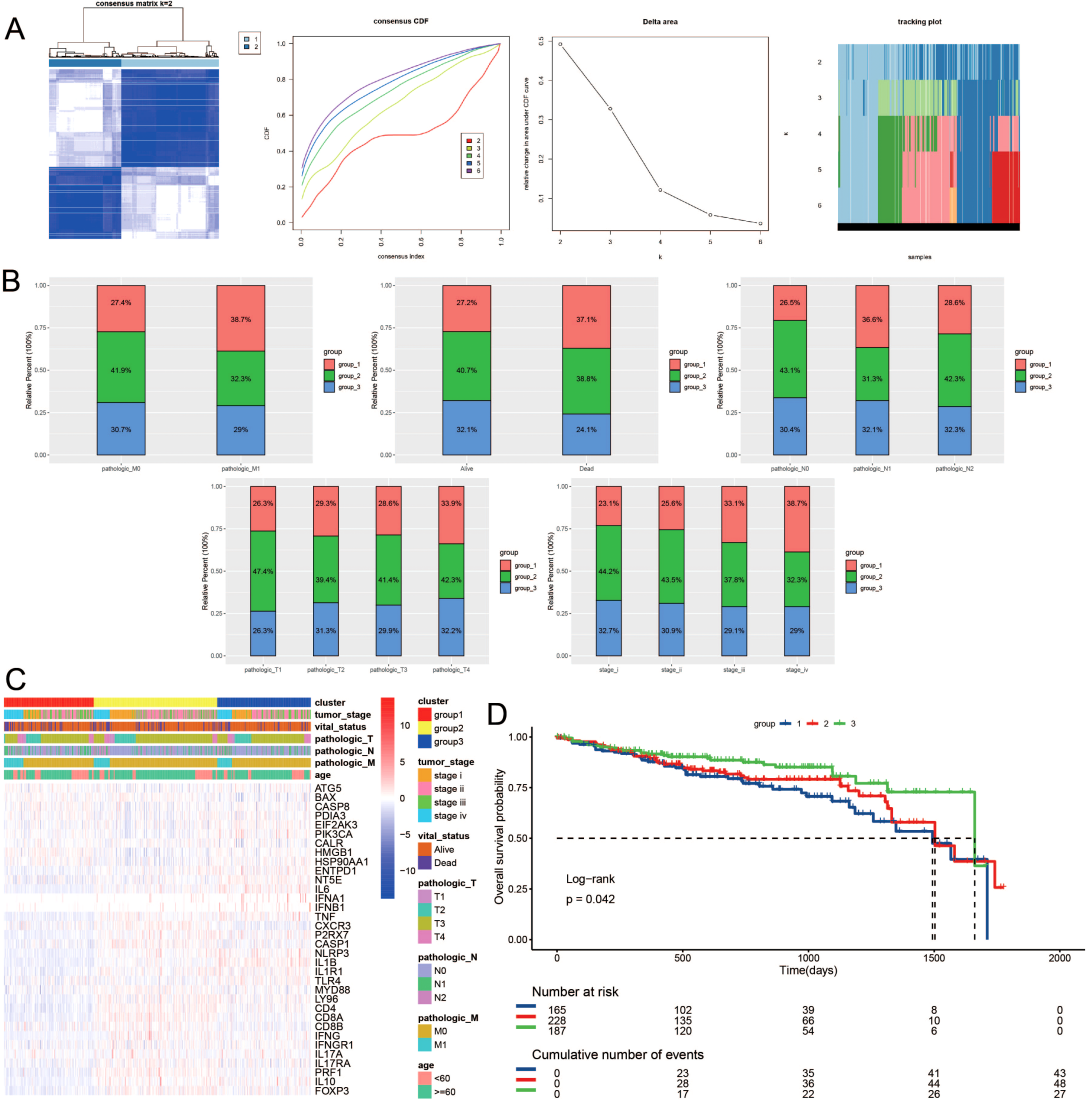
Consensus clustering of ICDRGs in CRC. The consensus matrices and **(A)** concensus CDF were performed to assess the stability of clustering and explore coagulation subtypes. **(B)** Distribution of clusters in clinical factors. **(C)** Unsupervised clustering heatmap of ICDRGs in TCGA cohorts. ICDRGs clusters, tumor stage, age, stage and survival state were used as patient annotations. **(D)** Prognosis analysis for the ICDRGs group1 and group2 across different stages of LUSC.

Building upon the observed expression differences of ICDRGs and their roles in various biological pathways, we further investigated whether these differences could define subtypes of CRC with distinct clinical outcomes. Utilizing unsupervised consensus clustering analysis, we identified distinct subtypes of TCGA colorectal cancer patients based on significantly different immunogenic cell death-related genes. Notably, when the survival curves indicated the presence of three subtypes, the patient survival analysis exhibited statistical significance (**Figure 2A**). **Figure 2B** depicts the categorization of colorectal cancer patients into three molecular subgroups, revealing varied distributions of molecular classifications alongside clinical indicators and follow-up data. Specifically, subtype 1 comprised 247 samples, subtype 2 had 222 samples, and subtype 3 contained 163 samples. Notably, a significant difference in survival outcomes was observed among the three subgroups, with subtype 3 exhibiting better survival prognosis and subtype 1 displaying poorer prognosis. Additionally, a higher proportion of patients with poor clinical staging was observed in subtype 1 (**Figures 2C, D**).

### Immune Cell Infiltration Evaluation

**Figure 3:**
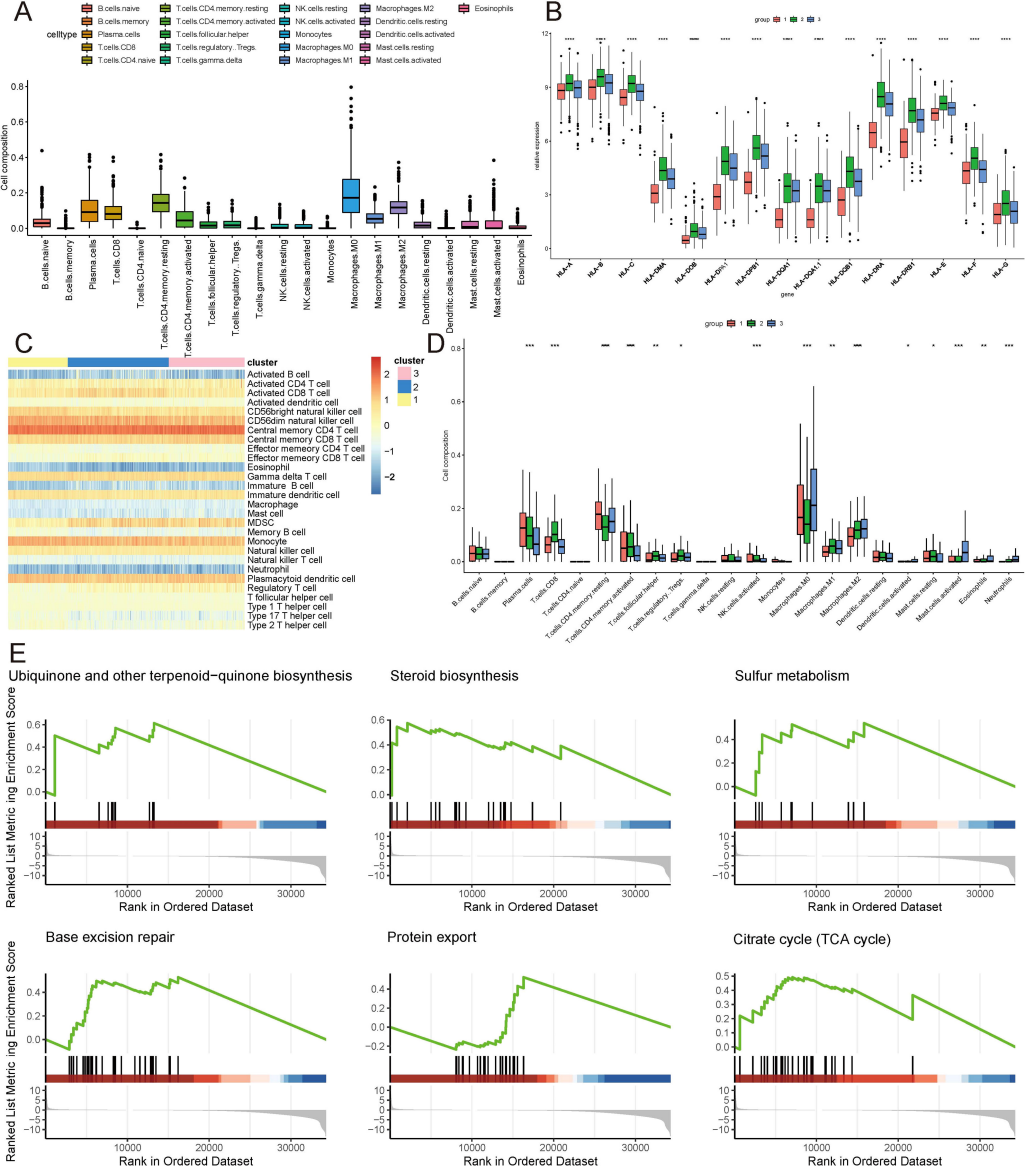
Immune landscape of ICDRGs cluster. **(A)** Immune cell infiltration, **(B)** Gene expression of HLA expression were analyzed between ICDRGs groups. **(C)** Ssgsea heatmap of immune cells. **(D)** A boxplot of the difference in immune cell infiltration in ICDRGs clusters. **(E)** ICDRGs cluster-based gsea analysis. Statistical significance at the level of * <0.05, ** <0.01, *** <0.001 and **** <0.0001.

Given the distinct subtypes identified and their differing survival outcomes, we explored the immune cell landscape within these subtypes to understand their immune microenvironment characteristics. Utilizing the CIBERSORT method, the 22 immune cell subtypes present in the TCGA samples were scrutinized within the three distinct subtype clusters. The analysis revealed significant infiltration of plasma cells, CD8+ T cells, resting and activated CD4+ memory T cells, follicular helper T cells, regulatory T cells, M0, M1, and M2 macrophages, activated dendritic cells, resting and activated mast cells, eosinophils, and neutrophils within these three subtype clusters, contributing substantially to the overall immune cell infiltration (**Figure 3A, D**). **Figure 3B** depicts the expression levels of HLA family genes across the three clusters, with cluster 1 exhibiting lower HLA gene expression. Complementing this, ssgsea analysis also indicated a similar pattern of immune cell infiltration (**Figure 3C**). Notably, GSEA revealed a prominent enrichment of isoforms involved in Ubiquinone and other terpenoid-quinone biosynthesis, Steroid biosynthesis, Sulfur metabolism, Base excision repair, Protein export, and the Citrate cycle (TCA cycle) (**Figure 3E**).

### Identification of tumor single cell landscapes and cell subsets associated with immunogenic cell death

**Figure 4:**
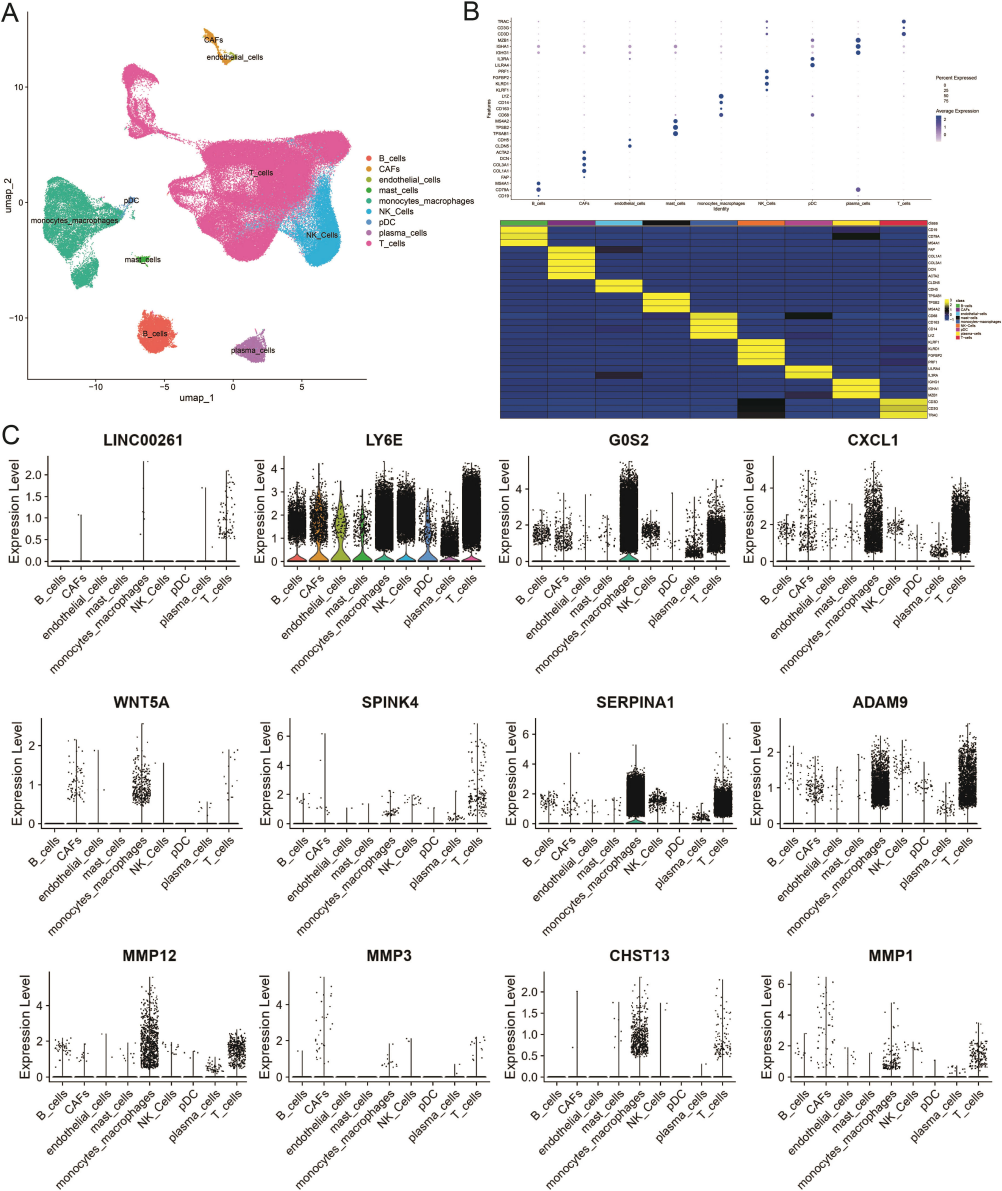
Single-cell atlas of primary CRC and liver metastases. **(A)** Overview of the workflow for single-cell transcriptome profiling of cells in primary CRC, matched liver metastases, and blood. **(B)** Heat map and dotplot showing differential expression of the top expressed genes in each cluster. For each cluster, the cell marker genes and their relative expression are shown. pDCs, plasmacytoid dendritic cells. **(C)** The violin diagram shows the subpopulation expression of ICDRGs in the single cell atlas.

To further unravel the cellular heterogeneity and explore the role of ICDRGs at the single-cell level, we analyzed a single-cell sequencing dataset, GSE178318, retrieved from the GEO database. The dataset comprises tumor tissues from 15 CRC patients. After integration and filtration of the raw data, the remaining cells and genes were utilized for subgroup identification and annotation. **Figure 4A** depicts the cellular subpopulation distribution among CRC patients. Employing the UMAP dimensionality reduction method, the expression patterns of individual cells were visualized based on the clustering results and cell marker annotations derived from the single-cell sequencing dataset. The results indicate that the cells can be classified into 28 subgroups, labeled as nine primary cell types (**Figure 4A**), ranging from 0 to 27. A heatmap was generated to illustrate the expression of the top two genes in each cellular subpopulation (**Supplementary Table S2**). A bubble plot further displays the expression and distribution of specific markers for each cellular subgroup (**Figure 4B**), highlighting the heterogeneity among tumor cells. Additionally, violin plots were utilized to demonstrate the distribution of ICDRGs across various cellular subpopulations (**Figure 4C**).

### Construction and validation of the prognostic model

**Figure 5:**
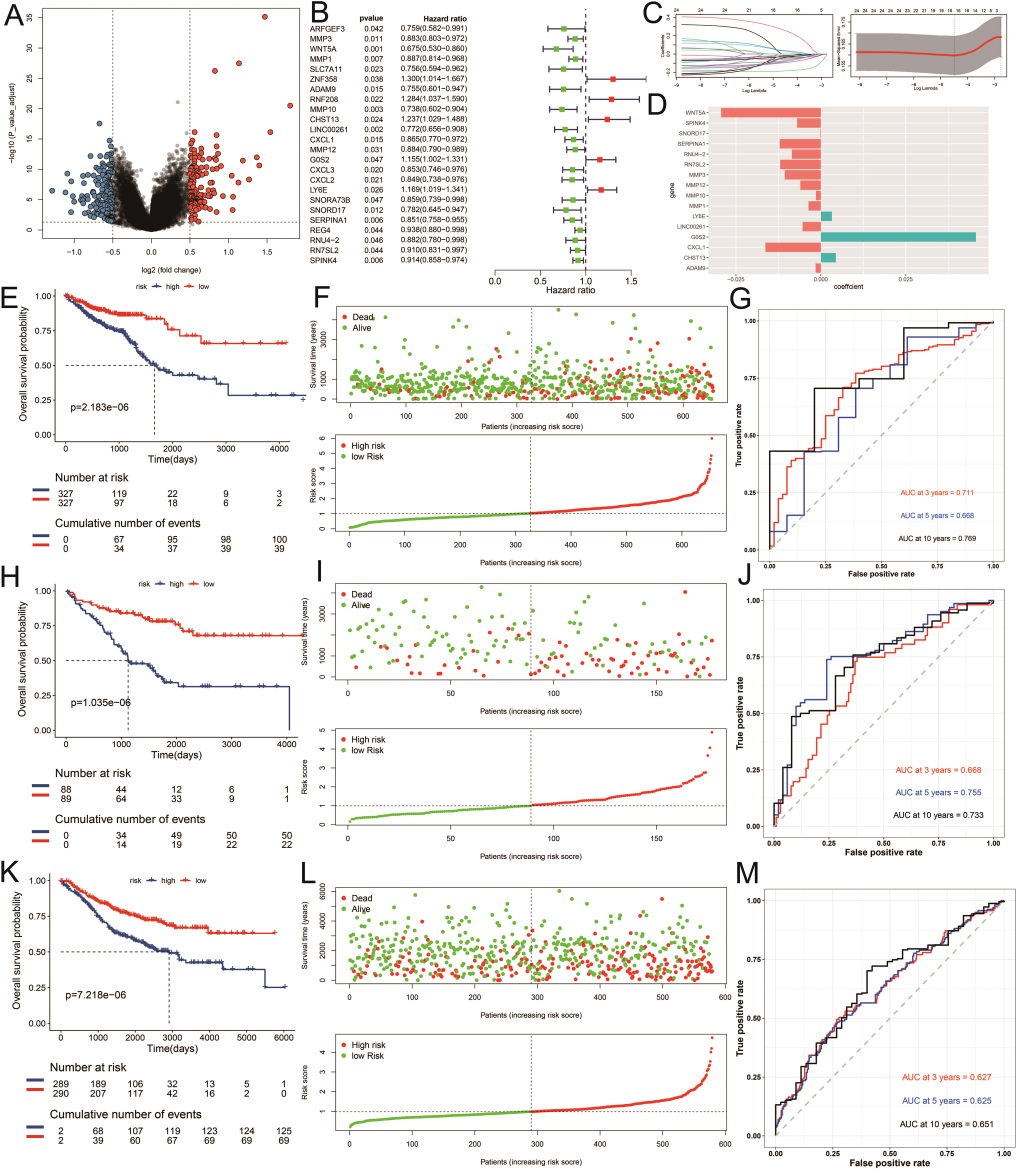
Construction of the risk model. **(A)** Differential expression analysis of volcanic maps. **(B)** Partial likelihood deviance was revealed by the LASSO regression model in the tenfold cross validation. **(C)** The vertical dotted lines were drawn at the optimal values by using the minimum and 1-SE criteria. **(D)** LASSO coefficient profiles of selected genes in the tenfold cross-validation. The vertical dotted lines were drawn at the optimal values by using the minimum criteria and 1-SE criteria. **(E, H, K)** Kaplan–Meier curves for the test and training sets, respectively (P < 0.001, log-rank test, survival rate comparison. **(F, I, L)** Patients were divided into high-risk and low risk subgroups based on median level of ICDRGs; survival status of patients in two subgroups. **(G, J, M)** Time-dependent receiver operating characteristic (ROC) of training and test sets.

Recognizing the clinical relevance of the identified ICDRGs, we moved on to construct a prognostic model to predict patient outcomes. We performed a differential gene expression analysis between tumor and normal samples, adopting a threshold of FDR (corrected p-value) less than 0.05 and an absolute log2 fold change (|log2FC|) greater than 1 to screen for significantly expressed genes. Among the differentially expressed genes across subtypes, we further identified 24 genes significantly associated with prognosis using univariate Cox regression analysis (P < 0.05) (**Figures 5A, B**). Utilizing the survival time and status data from the TCGA training set and two GEO validation sets (gse17536, gse39582), we employed the LASSO algorithm to identify and construct a risk model (**Figure 5C**). Ultimately, 16 genes were selected to build the risk scoring model (**Figure 5D**). Survival analysis revealed that a higher risk score correlated with poorer survival outcomes in both the training and testing groups (P < 0.05) (**Figures 5E, F, H, I, K, L**). The sensitivity of the model in predicting prognosis was assessed using time-dependent receiver operating characteristic (ROC) curves. The performance was evaluated based on the area under the ROC curve (AUC). For the training set, the 3-year, 5-year, and 10-year AUCs were 0.711, 0.668, and 0.769, respectively (**Figure 5G**). Similarly, for the testing set GSE17536, the AUCs were 0.668, 0.755, and 0.733 (**Figure 5J**), while for the testing set GSE39582, the AUCs were 0.627, 0.625, and 0.651 (**Figure 5M**). The time-dependent ROC curve analysis demonstrates that our prognostic model has solid predictive power, with AUC values of 0.711, 0.668, and 0.769 for 3, 5, and 10 years in the training set, respectively, indicating reliable discriminative ability for predicting colorectal cancer (CRC) patient survival. These results suggest that our model could be clinically valuable for risk stratification and personalized treatment decisions, offering accurate predictions for both short- and long-term outcomes.

### ICDRGs predict how chemotherapy works

**Figure 6:**
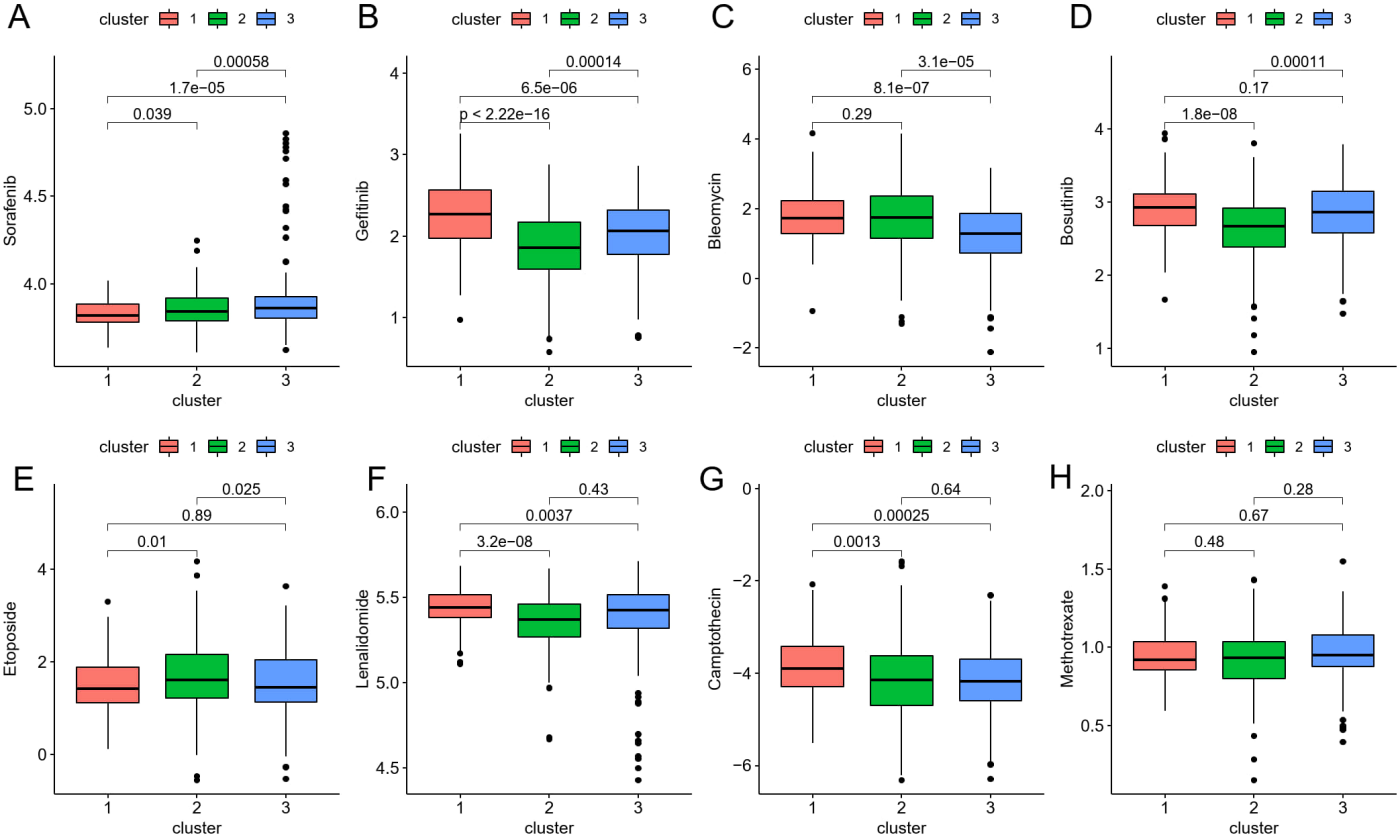
**(A–H)** The boxplot shows that drug sensitivity prediction score (Sorafenib, Gefitinib, Bleomycin, Bosutinib, Etoposide, Lenalidomide, Camptothecin, Methotrexate) are distributed differently among risk groups.

To translate these findings into therapeutic contexts, we next explored how the identified ICDRGs could predict the efficacy of chemotherapy in CRC patients. Eight chemotherapeutic medications’ IC50 discrepancies were investigated using the “pRRophetic” package to predict their sensitivity to drug therapy. The drug sensitivity data for Sorafenib, Gefitinib, Bleomycin, Bosutinib, Etoposide, Lenalidomide, Camptothecin, and Methotrexate in the CRC risk model were presented in **Figures 6A-H**, respectively. Among them, Gefitinib, Bosutinib, Etoposide, Lenalidomide, and Camptothecin had higher IC50 levels for cluster 1 patients with poor prognosis, providing certain guidance for the use of clinical chemotherapy drugs.

### Analysis of prognostic features of model genes

**Figure 7:**
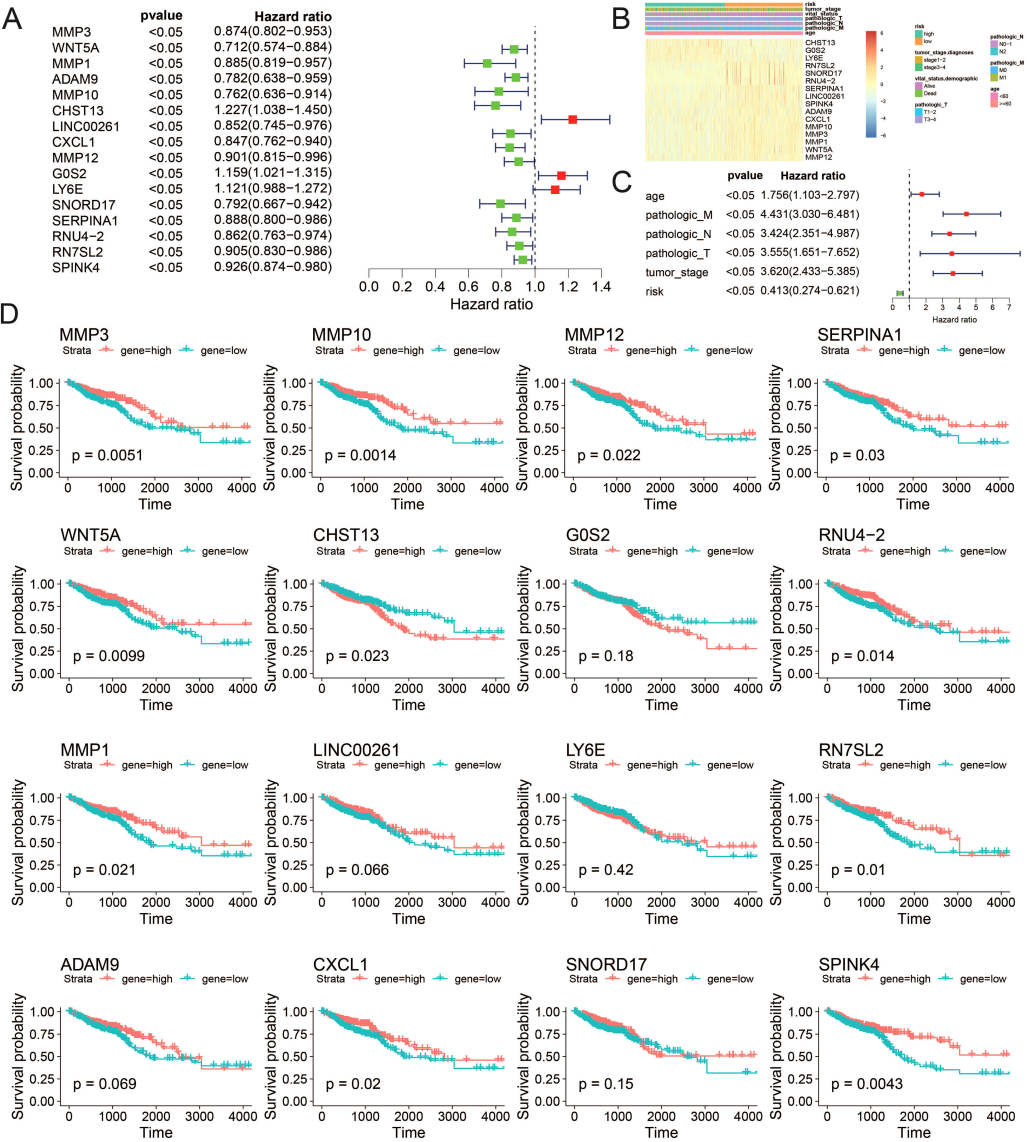
Correlation between the risk model and clinicopathological features of TCGA–CRC samples. **(A)** Univariate analysis of ICDRGs, **(B)** Gene expression heat maps and annotation of clinical data. **(C)** Multivariate analysis including ICDRGs, risk model and clinical factors. **(D)** Single gene survival analysis. ICDRGs.

Building on the chemotherapy sensitivity data, we further analyzed the prognostic features of the model genes to understand their impact on CRC patient outcomes. We employed single-factor Cox regression on the aforementioned LASSO regression model to identify genes significantly associated with prognosis (P < 0.05). In total, 16 genes were identified: MMP3, WNT5A, MMP1, ADAM9, MMP10, CHST13, LINC00261, CXCL1, MMP12, G0S2, LY6E, SNORD17, SERPINA1, RNU4-2, RN7SL2, and SPINK4 (**Figure 7A**). Subsequently, utilizing univariate Cox regression analysis, we separately examined the clinical factors including risk, TNM stage, age, and clinical stage, in accordance with the methodology outlined. This process allowed us to filter out variables with a p-value less than 0.05 (**Figures 7B, C**). The expression patterns of these 16 genes exhibited similarities to the distributions of clinical factors, indicating their potential impact on crucial clinical follow-up data. Moreover, survival analysis revealed that the expression of MMP1, MMP3, MMP10, MMP12, RNU4-2, SERPINA1, WNT5A, RN7SL2, CHST13, CXCL1, and SPINK4 significantly influenced patient survival (P value < 0.05) (**Figure 7D**).

### Gene expression level verification via quantitative reverse transcription PCR

**Figure 8:**
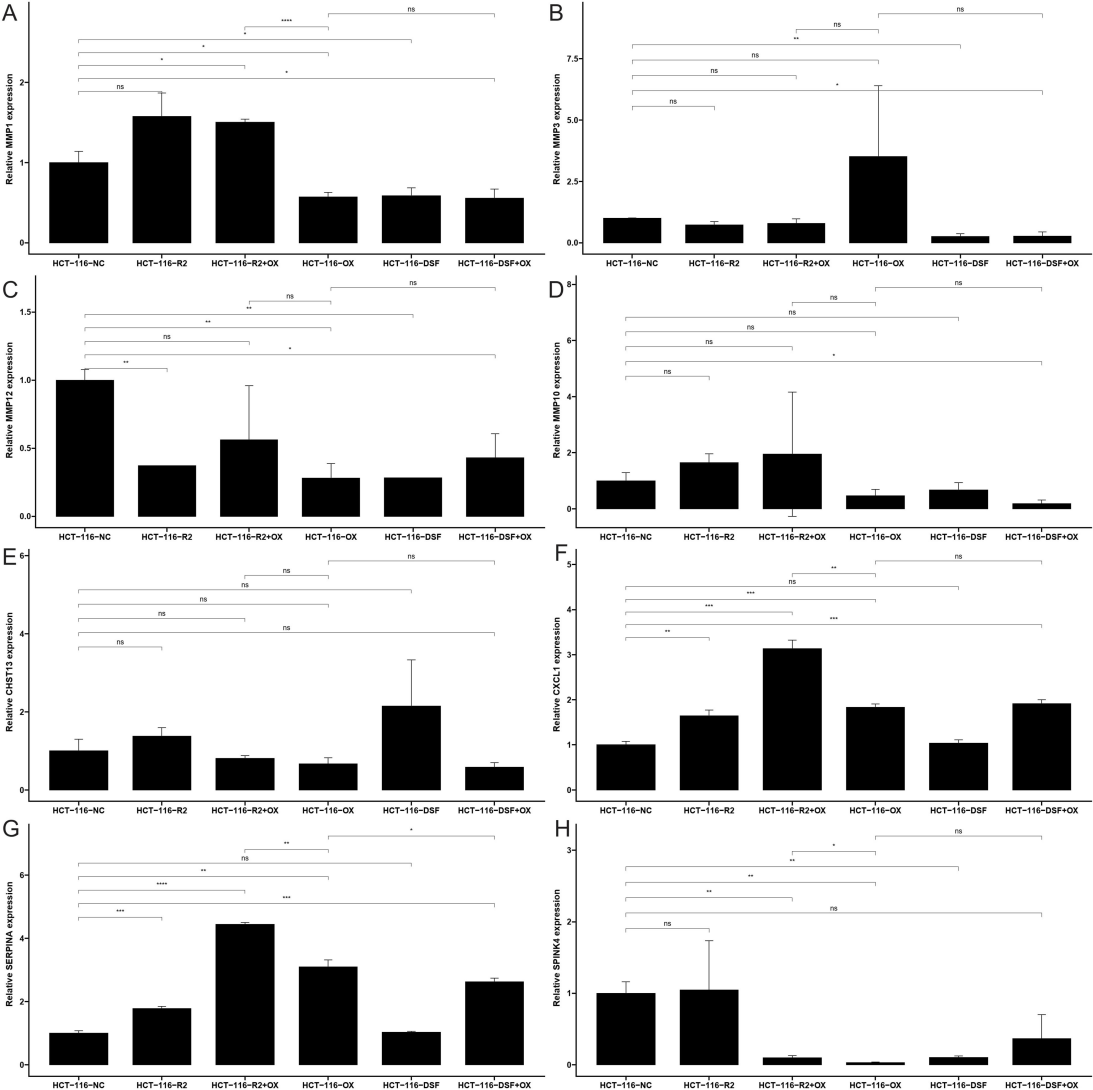
The mRNA expression of **(A)** MMP1, **(B)** MMP3, **(C)** MMP10, **(D)** MMP12, **(E)** CHST13, **(F)** CXCL1, **(G)** SERPINA1 and **(H)** SPINK4 in a CRC cell lines and the adjacent cell lines; ns: no significance, * <0.05, ** <0.01, *** <0.001 and **** <0.0001.

**Figure 9:**
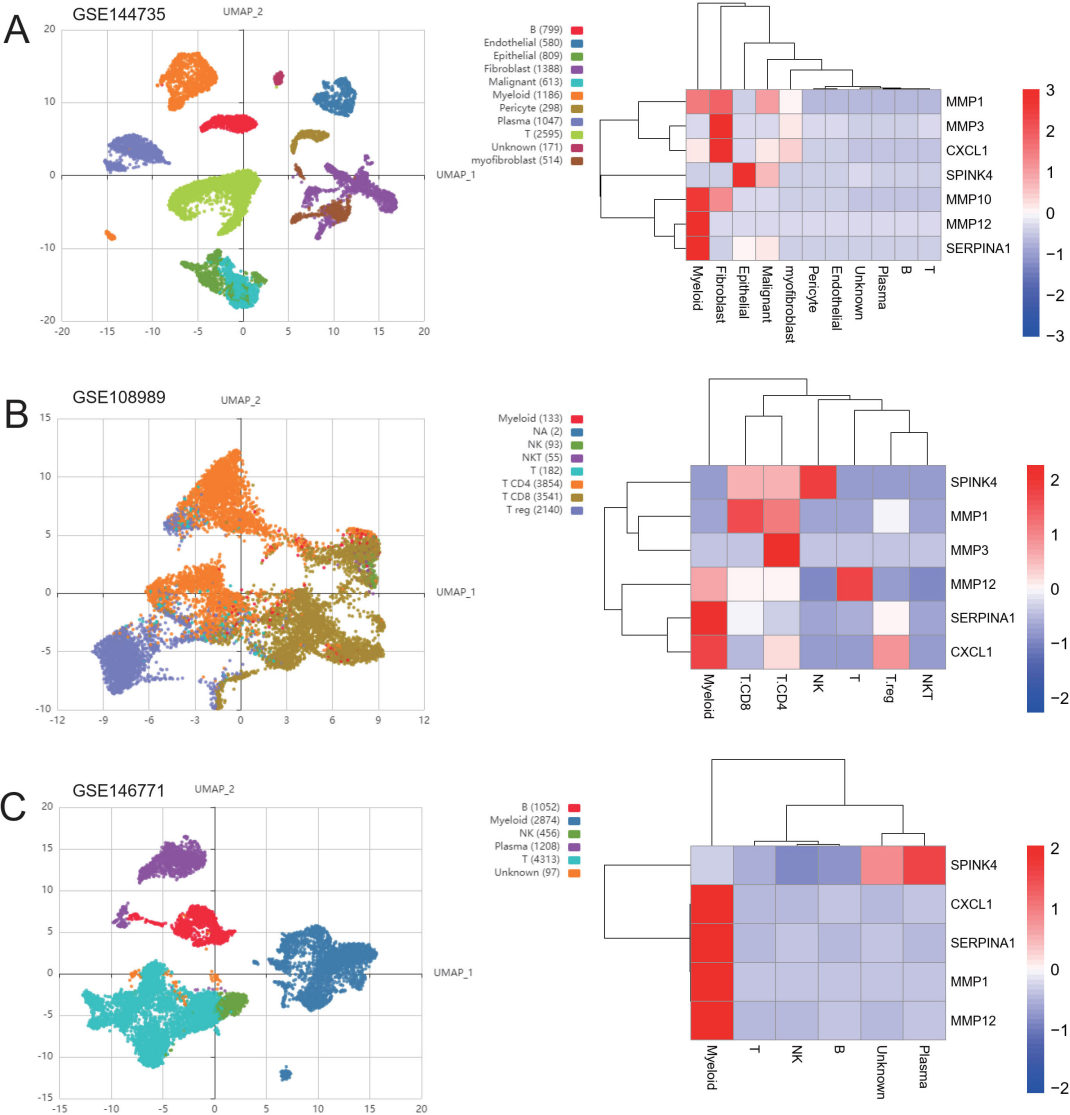
Distribution of genes in single-cell subsets. UMAP cluster map of the three single-cell data as well as the distribution map of cell subpopulation expression of ICDRGs. **(A)** GSE144735 **(B)** GSE108989 **(C)** GSE146771.

To validate the expression levels of the identified prognostic genes, we performed quantitative reverse transcription polymerase chain reaction (qRT-PCR) experiments. After incubating CRC cells for 12 to 24 hours, we investigated the changes in gene expression. In contrast to untreated cells, the expression of target genes significantly varied across different cell lines (**Figures 8A–H**). The cell lines used in this experiment showed consistent trends in gene expression levels, validating our computational predictions. **Supplementary Table S3** lists the sequences of primers utilized in this study. Additionally, we utilized public datasets to analyze the expression patterns of the 16 model genes across diverse clinical subgroups. The expression analysis results were consistent with the PCR validation data, corroborating the accuracy and reliability of our computational model (**Figures 8A–H**). Moreover, the prognostic risk model established based on the 16 identified ICDRGs successfully stratified patients into high-risk and low-risk groups. Higher risk scores correlated with poorer survival outcomes, as verified through external GEO datasets. This model provides a valuable tool for prognostic evaluation and risk stratification of CRC patients in clinical settings. Based on the analysis results of the three single-cell datasets, we found that SPINK4, MMP3 and CXCL1 were mainly distributed in immune T cells, while MMP1, MMP10, MMP12, and SERPINA1 were mainly distributed in Myeloid cell (**Figures 9A–C**).

## Discussion

Unlike the majority of non-immunogenic or even tolerogenic forms of normal cell apoptosis, ICD in cancer cells has the unique ability to elicit potent antitumor immune responses by activating antigen-presenting cells (such as dendritic cells, DCs) and subsequently stimulating cytotoxic T lymphocytes (CTLs) (27). This ICD process is orchestrated by the translocation of calreticulin (a “eat-me” signal for DC uptake) from the endoplasmic reticulum to the cell surface, concurrent with the release of HMGB-1 and ATP, which provide co-stimulatory signals to DCs (28). Recent research has demonstrated that select chemotherapeutic agents, radiotherapy, and photodynamic therapy can effectively induce ICD in cancer (29). Building upon this foundation, recent advancements in cancer immunotherapy have unveiled a promising approach to tackle the limitations of PD-1 checkpoint blockade in cold tumors through inducing ICD using novel agents such as ferroptosis inducers (10). Ferroptosis, an iron-dependent form of regulated cell death, mimics the immunogenic potential of ICD by triggering the release of damage-associated molecular patterns (DAMPs) (30). This strategy capitalizes on the potential of ferroptosis inducers to overcome the immunosuppressive tumor microenvironment by directly targeting tumor cells, causing them to undergo immunogenic death (31). By integrating these inducers with other therapeutic modalities, researchers have devised sophisticated systems that synergistically enhance ICD effects. For instance, a system combining polyethylene glycol (PEG)-functionalized polyphenols with ferroptosis-inducing agents and manganese dioxide nanoparticles (MnO2 NPs) has demonstrated remarkable potential (32). Upon systemic administration, MnO2 NPs decompose within the hypoxic tumor microenvironment, releasing oxygen and Mn2+ ions (33). This not only alleviates hypoxia but also facilitates ROS generation, which synergizes with the ferroptosis-inducing agents to potentiate ICD (34). The resulting cascade of events promotes the release of tumor-specific antigens, maturation of dendritic cells, and infiltration of tumor-specific T cells (35). This ICD-augmenting strategy initiates robust tumor-specific immune responses, activates antitumor immunity, and exhibits promising abscopal effects against distant tumors (36). The integration of ferroptosis inducers within such multimodal ICD-enhancing platforms holds significant promise for expanding the therapeutic landscape of chemotherapy-based ICD in the context of immune checkpoint blockade (37). By harnessing the immunogenic potential of ferroptosis, these approaches offer a novel avenue to overcome the challenges associated with PD-1 blockade in cold tumors, thereby enhancing overall treatment efficacy and patient outcomes (38).

Our study leverages comprehensive transcriptomic and clinical data from TCGA and GEO databases to explore the relationship between immunogenic cell death-related genes (ICDRGs) and colorectal cancer (CRC) progression, as well as their implications for clinical drug response. We identified 26 ICDRGs with distinct expression patterns in tumors, revealing a unique gene signature associated with CRC immunogenicity. Notably, the mutation frequencies of key ICDRGs, such as PI3KA, NLRP3, CASP8, and TLR4, highlight the potential role of genetic alterations in modulating immune responses within the tumor microenvironment. The unsupervised consensus clustering analysis further refined the CRC patient cohort into three distinct molecular subtypes, each exhibiting unique survival outcomes and clinical characteristics. Subtype 3 was associated with better survival prognosis. In contrast, Subtype 1 showed poorer prognosis and higher proportions of advanced clinical staging, underscoring the need for targeted therapeutic interventions.

The immune cell infiltration analysis, using CIBERSORT and ssGSEA, revealed profound immune cell heterogeneity across the molecular subtypes. There was notable infiltration of plasma cells, CD8+ T cells, and various macrophage subsets. This finding highlights the importance of immune cell composition in shaping the tumor immune landscape and its impact on patient outcomes. Additionally, the enrichment of biological pathways involved in inflammatory responses and immune signaling underscores the pivotal role of ICDRGs in modulating the immune response to CRC.

Our analysis of single-cell landscapes within CRC tumors offers unprecedented resolution into the cellular heterogeneity and ICDRG expression patterns. We identified 28 distinct cellular subpopulations, each with specific marker expression profiles. These findings provide a foundation for future studies aimed at dissecting the intricate interplay between tumor cells and the immune system.

The construction and validation of a prognostic model based on 16 differentially expressed genes significantly associated with CRC prognosis underscore the clinical utility of our findings. The model’s ability to accurately predict survival outcomes across independent datasets validates its robustness and generalizability. Moreover, the prediction of drug sensitivity based on ICDRG expression profiles offers valuable guidance for personalized chemotherapy regimens. Specifically, higher IC50 values were observed for Gefitinib, Bosutinib, Etoposide, Lenalidomide, and Camptothecin in the poor prognosis cluster, suggesting that alternative therapeutic strategies may be warranted for these patients.

The functional verification of gene expression changes following drug treatment, using qRT-PCR, strengthens our findings. It demonstrates the direct effects of RSL3, disulfiram, and oxaliplatin on ICDRG expression in CRC cells. The differential responses of MMP1, MMP3, MMP10, MMP12, SPINK4, SERPINA1, and CXCL1 to various drug treatments provide insights into the regulatory mechanisms underlying CRC drug resistance and sensitivity.

In summary, our study offers a comprehensive view of the complex interplay between ICDRGs, immune cell infiltration, and CRC progression. By identifying distinct molecular subtypes and prognostic gene signatures, we have paved the way for more targeted and personalized therapeutic approaches in CRC. Additionally, seven genes (MMP1, MMP3, MMP10, MMP12, SPINK4, SERPINA1, and CXCL1) were identified as related to patient survival and showed significant therapeutic effects in wet experiments. These genes can be used as effective targets for clinical treatment, and their expression levels can predict patient survival. Our findings on drug sensitivity prediction and gene expression changes under drug treatment offer valuable guidance for optimizing chemotherapy regimens and improving patient outcomes. These insights hold significant promise for advancing CRC clinical management and ultimately improving survival rates.

The application of ferroptosis inducers in CRC therapy presents groundbreaking potential by intricately modulating the tumor microenvironment. This is achieved through the regulation of genes such as MMP1, MMP3, MMP10, and MMP12, along with immune-related genes like SPINK4, SERPINA1, and CXCL1. This intricate network not only directly impedes tumor cell invasion and immune evasion but also indirectly triggers immunogenic cell death (ICD), mimicking natural immune activation signals. The release of damage-associated molecular patterns (DAMPs) like ATP and HMGB1 during ICD potently stimulates dendritic cells and promotes the infiltration of cytotoxic T lymphocytes, thereby bolstering antitumor immune responses. Moreover, these ICDRGs have a multifaceted role in reshaping the tumor microenvironment, distinguishing them from conventional therapeutic targets. For instance, matrix metalloproteinases (MMP1, MMP3, MMP10, MMP12), while traditionally associated with promoting tumor cell invasion and metastasis, have been shown in recent studies to facilitate ferroptosis in the right context by inducing lipid peroxidation. Lipid peroxidation is a critical mechanism of cell death in cancer therapy. The upregulation of these MMPs increases the production of lipid peroxides, directly promoting cancer cell death through ferroptosis. Additionally, these MMPs help regulate cytokine release during ICD, leading to enhanced infiltration of immune cells, such as dendritic cells (DCs) and cytotoxic T lymphocytes (CTLs). This further intensifies the antitumor immune response. This dual capability of MMPs to promote direct cancer cell death and enhance the immune response underlines their potential as a novel therapeutic target distinct from conventional approaches.

Similarly, immune-related genes such as SPINK4, SERPINA1, and CXCL1 play crucial roles in modulating immune responses. CXCL1, for example, is a chemokine that attracts neutrophils and other immune effector cells to the tumor site, amplifying immune cell-mediated cytotoxicity. By incorporating these genes into ferroptosis-inducing strategies, the tumor microenvironment can be made more immunogenic. This process effectively transforms it from a tumor-promoting to a tumor-suppressive state. These effects are particularly relevant in CRC, where immune evasion remains a significant challenge.

Furthermore, ferroptosis inducers have been shown to significantly impact macrophages, which are pivotal players in the immune system and the tumor microenvironment (39). Macrophages, versatile cells capable of phagocytosing apoptotic and necrotic debris while secreting both pro- and anti-inflammatory cytokines, undergo significant alterations under the influence of ferroptosis inducers. Their activation leads to enhanced phagocytosis of tumor fragments and the release of proinflammatory cytokines like TNF-α and IL-1β. This further intensifies antitumor immunity. The preferential expansion of M1 macrophages, known for their superior antigen-presenting capabilities and tumoricidal effects, underscores the potential of ferroptosis inducers to skew the immune landscape toward a more tumor-hostile state.

Building on this, Mau-Shin Chi et al. have highlighted that while traditional ferroptosis inducers, such as conventional radiotherapy, chemotherapeutic drugs, and hyperthermia, often fail to elicit a robust immune response in clinical settings, newer ferroptosis inducers show more promise. These novel agents include various nanoparticles (NPs), sparse-focused radiotherapy (SFRT), magnetic particle thermotherapy (MPT), and low-frequency radiofrequency hyperthermia. They possess the unique ability to induce ferroptosis heterogeneously within tumors, either randomly or selectively. The study suggests that this uneven distribution of ferroptosis might not be a disadvantage. Instead, the peak-to-valley distribution within the tumor could be a viable strategy to enhance the efficacy of tissue-resident memory T cells (TRM) in cancer therapy. Combining NPs with conventional radiotherapy or low-frequency radiofrequency hyperthermia could sensitize these treatments. This makes the tumor microenvironment more immunogenic and enhances therapeutic outcomes. These insights suggest that the therapeutic potential of ferroptosis inducers lies not only in their direct effects on tumor cells but also in their ability to modulate the immune microenvironment in innovative ways.

For CRC patients who are unresponsive to conventional therapies, ferroptosis inducers offer a promising avenue that could synergize with existing immunotherapies, such as PD-1 blockade, to achieve more precise and effective personalized treatment strategies. The combination of ferroptosis inducers and immunotherapies could therefore unlock new opportunities for advancing CRC clinical management and improving patient outcomes. By harnessing the power of ferroptosis inducers, particularly those targeting ICDRGs like MMP1, MMP3, MMP10, MMP12, SPINK4, SERPINA1, and CXCL1, we may finally realize the full potential of the immune system in the fight against CRC. The ability of these novel agents to modulate both the tumor and immune landscapes marks a significant advancement over existing targets, providing a dual approach that is both directly cytotoxic to cancer cells and capable of reprogramming the immune microenvironment for sustained therapeutic effects.

Undoubtedly, our study is subject to certain limitations that warrant acknowledgment. Firstly, given the heterogeneity of our study cohort, which comprises data sourced from diverse high-throughput sequencing platforms and public datasets, intratumoral and interpatient tumor heterogeneity is an inevitable factor. Several studies have highlighted the impact of tumor heterogeneity on the efficacy of immunotherapy and chemotherapy. Unfortunately, due to data constraints, we had to overlook the significant heterogeneity inherent in CRC, which could potentially influence our findings. Secondly, although our study identified immune interactions and survival impacts involving inflammatory pathways and ICD targets in CRC patients, the underlying biological or medical mechanisms remain elusive. Thus, large-scale prospective studies complemented by functional and mechanistic experiments are imperative to validate and elucidate the role of inflammatory pathways in CRC. Thirdly, the median cutoff value of survival-associated ICD genes was employed to stratify CRC samples into high- and low-survival groups. However, it is plausible that an optimized cutoff value tailored specifically for survival-related ICD genes could offer a more refined stratification strategy for CRC patients. Lastly, owing to the scarcity of comprehensive clinicopathological information, we had to curate and adjust certain clinical data for survival and Cox regression analyses. This approach, while necessary, might introduce potential biases and uncertainties regarding the independence of ICD-related groups as prognostic factors. While our study provides valuable insights, future endeavors should aim to address these limitations through more rigorous data collection, analysis, and experimental validation, ultimately contributing to a deeper understanding of CRC and the development of more effective therapeutic strategies.

## Conclusion

Our comprehensive study has unveiled a set of key immunogenic cell death-related genes (ICDRGs) that serve as potential targets for clinical ferroptosis-inducing therapies in colorectal cancer (CRC). By leveraging transcriptomic and clinical data, we identified these ICDRGs, as crucial players in modulating the tumor immune microenvironment and CRC progression. The distinct expression patterns and mutation frequencies of these targets highlight their suitability for targeted intervention. Our analysis further underscores the potential of these ICDRGs to enhance the efficacy of ferroptosis-based treatments by selectively inducing tumor cell death while stimulating anti-tumor immune responses. The development of therapies targeting these ICDRGs as clinical ferroptosis inducers holds significant promise for improving CRC patient outcomes and advancing clinical management strategies.

## Data Availability

Publicly available datasets were analyzed in this study. This data can be found here: https://portal.gdc.cancer.gov/ and https://www.ncbi.nlm.nih.gov/. The names of the repository/repositories and accession number(s) can be found in the article/**Supplementary Material**.
